# How to optimize the systematic review process using AI tools

**DOI:** 10.1002/jcv2.12234

**Published:** 2024-04-23

**Authors:** Nicholas Fabiano, Arnav Gupta, Nishaant Bhambra, Brandon Luu, Stanley Wong, Muhammad Maaz, Jess G. Fiedorowicz, Andrew L. Smith, Marco Solmi

**Affiliations:** ^1^ Department of Psychiatry University of Ottawa Ottawa Ontario Canada; ^2^ Department of Medicine University of Calgary Calgary Alberta Canada; ^3^ College of Public Health Kent State University Kent Ohio USA; ^4^ Department of Family Medicine University of Ottawa Ottawa Ontario Canada; ^5^ Department of Medicine University of Toronto Toronto Ontario Canada; ^6^ Department of Psychiatry University of Toronto Toronto Ontario Canada; ^7^ Faculty of Medicine University of Toronto Toronto Ontario Canada; ^8^ Department of Mechanical and Industrial Engineering University of Toronto Toronto Ontario Canada; ^9^ Department of Mental Health The Ottawa Hospital Ottawa Ontario Canada; ^10^ Ottawa Hospital Research Institute (OHRI) Clinical Epidemiology Program University of Ottawa Ottawa Ontario Canada; ^11^ School of Epidemiology and Public Health Faculty of Medicine University of Ottawa Ottawa Ontario Canada; ^12^ Department of Child and Adolescent Psychiatry Charité ‐ Universitätsmedizin Berlin Berlin Germany

**Keywords:** artificial intelligence, ChatGPT, evidence synthesis, large‐language models, systematic review

## Abstract

Systematic reviews are a cornerstone for synthesizing the available evidence on a given topic. They simultaneously allow for gaps in the literature to be identified and provide direction for future research. However, due to the ever‐increasing volume and complexity of the available literature, traditional methods for conducting systematic reviews are less efficient and more time‐consuming. Numerous artificial intelligence (AI) tools are being released with the potential to optimize efficiency in academic writing and assist with various stages of the systematic review process including developing and refining search strategies, screening titles and abstracts for inclusion or exclusion criteria, extracting essential data from studies and summarizing findings. Therefore, in this article we provide an overview of the currently available tools and how they can be incorporated into the systematic review process to improve efficiency and quality of research synthesis. We emphasize that authors must report all AI tools that have been used at each stage to ensure replicability as part of reporting in methods.

**Key points**

**What's known**

- Systematic reviews are an essential cornerstone for synthesizing available evidence on a given topic or research question.
- Numerous artificial intelligence (AI) tools are being released weekly, many of which are geared toward optimizing efficiency of academic writing.

**What's new**

- Given the large number of AI tools available, it can be challenging for researchers to determine which tools are most appropriate and effective for their specific research question or project. We provide an overview of the currently available tools and how they can be incorporated into the systematic review process.

**What's relevant**

- The effective integration of AI tools into the systematic review process has considerable potential to significantly improve efficiency and streamline the research workflow, while accelerating development of new, more targeted tools.

## BACKGROUND

Systematic reviews are an essential cornerstone for synthesizing the available evidence on a given topic or clinical or research question. They simultaneously enable gaps in the literature to be identified and provide direction for further research. However, as the volume and complexity of the available literature continue to grow exponentially, traditional methods for conducting systematic reviews are becoming less efficient and more time‐consuming.

The methodology of a systematic review can be summarized into five main steps (Khan et al., 2003). First, one must frame the research question which must be clear and developed prior to beginning the review work itself. Second, relevant studies must be found through a systematic search of multiple databases by at least two authors. The selection of studies for inclusion must flow directly from the research question and specified a priori, with any discrepancies noted. Third, a quality assessment of all included studies must be conducted. This can be accomplished through readily available critical appraisal guides, which are often in the form of checklists. This is crucial in order to assess the reliability of inferences drawn from pertinent findings. Fourth, the evidence gathered from the search must be summarized. Depending on study type and heterogeneity, this can either be accomplished narratively or by combining effects by means of meta‐analysis. Lastly, the findings of the study must be interpreted. To do this, one must explore explanations for their findings and the relevance that they may have to practice. One could consider making recommendations based on their findings, however this should be contextualized based on the strengths and weaknesses of the evidence available.

Particularly, in the field of mental health and psychiatry, high‐quality systematic reviews are crucial (Bellato, Cristea, et al., 2023). These systematic reviews integrate evidence from various sources, providing comprehensive overviews of the available literature for a given topic. This allows for informed decisions to be made by psychiatrists and gaps in the literature to be unveiled, providing direction for future research. In psychiatry, elements such as early predictors of psychopathology, disease prognosis and effectiveness of interventions are of particular interest to clinicians as they assist in determining appropriate treatment regimes (Bellato, Admani, et al., 2023; Fabiano et al., 2023; Solmi et al., 2023). Oftentimes, individual primary studies may either be underpowered to detect a particular outcome of interest or have conflicting outcomes based on other available literature. In these instances, it is imperative that high‐quality evidence synthesis studies are available in order to unify these findings to inform clinical practice.

On average, it takes 67 weeks and considerable human resources to take a systematic review from protocol registration to publication, with a range from 6 months to as long as 3 years, depending on the scope, methodology and resources available (Borah et al., 2017; Dicks et al., 2014). Psychiatry and mental health researchers may face unique challenges in conducting systematic reviews, compared to colleagues in other disciplines. Particularly, mental health research is affected by heterogeneous diagnostic criteria, subjective outcomes, confounding factors, episodic or continuous disease course, complex etiology, long duration of untreated illness, frequent diagnostic migration, which require that all studies potentially eligible for systematic reviews are carefully scrutinized from researchers with clinical and methodological expertise, thereby increasing the time required to complete a review (Kingdon, 2006). By incorporating artificial intelligence (AI) tools into the systematic review process, authors have claimed this timeline can be significantly reduced to an impressively short 2 weeks (Clark et al., 2020). However, these fast timelines should be considered with caution, to ensure that study quality is not negatively influenced as a result. AI tools have the potential to automate many of the routine and time‐consuming processes involved in traditional manual reviews, and to also assist with the writing and editing of the final manuscript, with unprecedented efficiency in the complex and numerous steps of systematic reviews, but at the same time with associated risks. This has led some to suggest the use of machine learning tools in research synthesis (Marshall & Wallace, 2019).

Numerous AI tools are being released weekly, many of which are geared toward optimizing efficiency and academic writing (Golan et al., 2023). These tools have the potential to assist with various stages of the systematic review process including developing and refining search strategies, screening titles and abstracts for inclusion or exclusion criteria, extracting essential data from studies and summarizing findings. However, these tools should not be utilized as a substitute for human expertise and judgment. Quality and ethical risks are associated with the use of AI in evidence synthesis. Indeed, researchers should view AI as a supplementary tool that can help streamline and optimize the research process, while still allowing for critical quality check, human analysis, evaluation, and interpretation, and ultimately substantial contribution to each scientific report to meet International Committee of Medical Journal Editors criteria for authorship of any scientific report resulting from processes involving or mediated by AI.

Given the large number of AI tools currently available, it can be challenging for researchers to determine which tools are the most appropriate and effective for their specific research question or project. Therefore, the objective of this article is to provide an overview of some currently available tools and how they can be incorporated into the systematic review process to improve efficiency and quality of research synthesis. We also discuss responsible use and reporting of such assistance.

## INTRODUCTION TO AI

The cornerstone of modern automation of the systematic review process depends on the AI capabilities of large‐language models (LLMs), such as OpenAI's gpt3 or gpt4 (Introducing ChatGPT, n.d.). LLMs are models that have been trained on very large datasets of text, in order to demonstrate comprehension of the provided text. This section serves as an introduction to LLMs, however it is important to note that not all tools discussed are primarily based on this approach.

LLMs primarily use the Transformers‐based architecture, first developed in 2017 (Vaswani et al., 2017). This architecture uses “tokens” as pieces of natural text to be focused on via the Self‐Attention mechanism (Vaswani et al., 2017). By considering the relation between words in the same sentence, the self‐attention mechanism allows the model to focus on more salient words in a sentence (Vaswani et al., 2017). For an LLM to predict the word “tail” at the end of “The dog chased its tail,” the self‐attention mechanism will focus most on words that are most important, such as “dog” and “chased” (Vaswani et al., 2017). This feature, along with positional encoding to represent the position of words in a sentence and other technical features, made Transformers a crucial advance in the development of LLMs and the ability of machines to comprehend free‐flowing text (Vaswani et al., 2017). The subsequent training of these architectures on vast amounts of natural language has allowed for the development of these LLMs, which are able to comprehend a wide variety of user input and provide a relevant response (Radford et al., 2019).

As the systematic review screening process relies on understanding, comprehending, and digesting large amounts of text, most systematic review tools utilize LLMs for their analysis. Many tools available for the automation of the systematic review process utilize OpenAI's ChatGPT application programming interface (API) in order to utilize their LLM in novel and inventive ways to assist the systematic review process (OpenAI, 2023). For example, ChatPDF allows users to upload PDFs, which are then entered as text input to the ChatGPT API (ChatPDF ‐ Chat with Any PDF!, n.d.; Lichtenberger, 2023). This allows the user to then query the PDF using natural language, allowing for the user to ask questions such as “Is this study a randomized‐control trial (RCT)?.” ChatPDF, by utilizing the GPT‐3.5 API, can then output whether the PDF contains a RCT, and respond to the user in natural text (Lichtenberger, 2023).

ChatGPT can also be used to develop research questions. For example, formulating the research question may occur by prompting ChatGPT to “Define a research question for a systematic review comparing the efficacy of two drugs,” and then subsequently prompt it to “Define a search strategy for this research question” (OpenAI, 2023). As ChatGPT relies on text input and output, tasks such as defining a search strategy can be specified to be outputted in a format that contains the conditional logic for use in systematic review databases as well (OpenAI, 2023).

### AI tools available for the systematic review process

The systematic review process can be segmented into several stages, each presenting unique challenges that AI tools can help address. However, it is crucial to recognize that the landscape of AI tools is rapidly evolving, and this overview serves as a framework for how to incorporate such tools rather than a comprehensive or definitive guide. There is a diverse collection of AI tools which have been released to help expedite the systematic review process which are summarized in Tables 1 and 2. Below we describe the capabilities of each tool and propose how they can be incorporated to improve efficiency.

**TABLE 1:** AI tools to optimize the systematic review process.

Section	AI tool	URL	Description
Abstract	Graphical Abstract Maker	https://mindthegraph.com/app/graphical-abstract-maker	• Simplifies complex research into understandable visuals.
Introduction	Elicit.org	https://elicit.org/	• Assists in formulating well‐thought research questions during the early phases of a systematic review which can help to brainstorm a research question.
	OpenAI ChatGPT	https://chat.openai.com/	• Refines research questions by providing diverse perspectives and structured approaches, enhancing the brainstorming process.
			• Summarizes text data into concise, meaningful abstracts, thus saving time
			• Helps in structuring the introduction by generating suggestions based on provided information.
	ForeFront AI Chat	https://chat.forefront.ai/	• Allows the creation of unique writing personas and writing voices, enhancing the manuscript's style and tone.
	Scite.aI	https://scite.ai/	• Finds citations related to GPT‐generated responses, allowing for the user to find relevant sources to cite.
	Jenni.aI	https://jenni.ai/	• Facilitates the writing process by offering prompts and suggesting text, thereby making it easier to write the rationale.
	Research Rabbit App	https://researchrabbitapp.com/	• Suggests related papers based on Zotero saved ones, promoting thorough research for the rationale.
	Consensus.app	https://consensus.app/	• Simplifies the research process by summarizing top papers and consensus from the scientific community, which can be used in the rationale.
	EvidenceHunt	https://evidencehunt.com/	• Generates answers with cited articles, facilitating a swift and efficient research process for the rationale.
	LitSuggest	https://www.ncbi.nlm.nih.gov/research/litsuggest/	• Recommends similar studies based on your research interest using machine learning.
	PaperDigest	https://www.paperdigest.org/	• Assists in building a solid literature review on any topic, providing a strong foundation for the rationale of the study and existing state of the literature.
Methods	Distiller SR	https://www.distillersr.com/products/distillersr-systematic-review-software	• A validated AI tool performs literature screening after training on a proportion of hits
	Xtrct	http://xtrct.app	• Employs semantic search to filter for eligibility criteria, thereby enhancing study selection efficiency.
			• Finds at least five articles for MeSH terms, facilitating a targeted and specific search strategy.
	WiseOne.io	https://app.wiseone.io/	• Ensures information reliability by presenting various sources discussing the same subject.
	SearchSmart	https://www.searchsmart.org/	• Assists in selecting the best research database, maximizing the relevance and quality of search results.
	CitationChaser	https://estech.shinyapps.io/citationchaser/	• Identifies relevant research topics by tracing citation networks, thus expanding the scope of the search strategy.
	Consensus.app	https://consensus.app/	• Searches for relevant literature based on any question, ensuring a thorough search strategy.
	PaperDigest	https://www.paperdigest.org/	• Aids in constructing a comprehensive literature review, ensuring a solid understanding of the research field for the search strategy.
	Thalia	http://nactem-copious.man.ac.uk/Thalia/	• Enhances the efficiency of search results by allowing specific concept searches in PubMed.
	Covidence	https://www.covidence.org/	• Ranks articles by relevance using an active learning variant, enhancing the efficiency of the selection process.
	RCT Tagger	http://arrowsmith.psych.uic.edu/cgi-bin/arrowsmith_uic/RCT_Tagger.cgi	• Estimates the likelihood of PubMed articles being RCTs, thereby distinguishing between RCTs and non‐RCTs accurately.
	Rayyan.ai	https://www.rayyan.ai/	• Uses exclusion criteria to automatically identify articles for exclusion, streamlining the selection process.
	ChatPDF	https://www.chatpdf.com/	• Extracts data from research papers, though its accuracy should be manually verified due to its unvalidated status.
	RobotReviewer	https://www.robotreviewer.net/	• Expedites the data collection process by automatically extracting data regarding the trial conduct (i.e, the 'PICO', study design and risk of bias) from research papers.
			• Provides an automatic bias assessment in RCTs, recommended for semi‐automatic use to verify accuracy.
	PDFGear	https://www.pdfgear.com/	• Extracts text and facilitates interactions with research papers by answering user's queries.
	Humata.ai	https://www.humata.ai/	• Allows users to interactively query about research papers, enhancing comprehension of the content.
	HeyGPT.Chat	https://heygpt.chat/	• Enables interactive conversations to query uploaded research papers.
	OpenAI ChatGPT	https://chat.openai.com/	• Assists in data analysis tasks, for example, generating code for statistical analysis in R or Python.
Results	HeyGPT.Chat	https://heygpt.chat/	• Interprets uploaded research papers and answers user's specific queries, for instance, about potential biases in studies.
	OpenAI ChatGPT	https://chat.openai.com/	• Assists in data analysis tasks, for example, generating code for statistical analysis in R or Python which can be used in result synthesis.
Discussion	Scite.ai	https://scite.ai/	• Finds citations related to GPT‐generated responses, allowing for the user to find relevant sources to cite.
	Jenni.ai	https://jenni.ai/	• Stimulates the writing process by providing prompts and suggesting text, enriching the discussion section.
	OpenAI ChatGPT	https://chat.openai.com/	• Used to generate counterpoints and arguments for the discussion, ensuring a balanced analysis.
	CitationChaser	https://estech.shinyapps.io/citationchaser/	• Deepens the understanding of related topics by tracking citations, providing substance for the discussion.
	Consensus.app	https://consensus.app/	• Explores a topic thoroughly by searching for relevant literature to be used as citation sources.
	Scholarcy	https://www.scholarcy.com/	• Quickly summarizes any manuscript which can assist in the writing of a discussion section.

**TABLE 2:** Correlation matrix of tools and respective manuscript sections.

	Protocol		Screening/data extraction		Manuscript				
Tools	Research question	Search key	Screening	Data extraction	Introduction	Abstract	Methods	Results	Discussion
ChatPDF							✓	✓	
CitationChaser		✓					✓		✓
Consensus.app							✓		✓
Covidence			✓						
Distiller SR			✓						
Elicit.org					✓				
EvidenceHunt					✓				
ForeFront AI Chat					✓				✓
Graphical Abstract Maker						✓			
HeyGPT.Chat							✓	✓	
Humata.ai							✓	✓	
Jenni.ai					✓				✓
LitSuggest					✓				
OpenAI ChatGPT	✓				✓	✓	✓	✓	
PaperDigest		✓			✓		✓		
PDFGear							✓	✓	
Rayyan.ai			✓						
RCT Tagger							✓	✓	
Research Rabbit App					✓				
RobotReviewer							✓	✓	
Scholarcy									✓
Scite.ai					✓				✓
SearchSmart							✓		
Thalia		✓					✓		
WiseOne.io		✓					✓		
Xtrct		✓					✓	✓	

### Introduction

#### Formulating the research question

The key focus of a systematic review revolves around a clear, achievable, and novel research question. AI tools can aid in this by helping the user brainstorm and generate ideas, which they can adapt. For example, OpenAI ChatGPT can be prompted to generate research ideas on specific topics, and with web browsing capability can perform a rudimentary online search (OpenAI, 2023).

#### Creating the title

The title is an essential piece of any article. It is the first thing that readers will see and should convey the most important aspects of a paper to engage with them. Indeed, the first study screening in systematic reviews is at title/abstract level, reflecting the importance of an informative title to communicate the content and focus to readers. AI tools such as Elicit.org can help in the formulation of a well‐designed research question that will become the pillarstone of designing an impactful title (Ought, 2023). The research question generated from Elicit.org can then be used into OpenAI ChatGPT to be prompted to generate multiple titles for the article based on the tone, audience, and emphasis on key phrases.

#### Writing the abstract

The abstract provides readers with a concise summary of the article's rationale, methodology, results, and conclusions. However, the heterogeneity of abstract submission guidelines can create limitations that can restrict how much information can be conveyed.

AI tools can assist in creating abstracts within the set parameters of a journal, allow you to provide it with as much information as possible, and condense this information into an easy to read and publisher friendly format. For example, for written abstracts, OpenAI ChatGPT can be prompted to create an abstract of the information that is provided to it and generate pertinent keywords for the article. Alternatively, for graphical abstracts, Mind the Graph's Graphical Abstract Maker can propose an initial graphical poster from any article or conference abstract (Pamplona & Minozzo, 2022). Of course, human input is still required to both structure and verify the content of the abstract at this stage.

#### Writing

The introduction section sets the context for the review, reviews existing literature, and describes the importance of the project. AI tools can aid in the efficiency of writing the introduction section, such as by correcting grammatical errors, optimizing English language, avoiding repetitions, and making the text more or less discoursive. For example, Jenni.ai aids in this process by correcting grammar, analyzing text to make suggestions, and also suggesting condensed/summarized information to reduce word count and help make writing more concise (Park et al., 2019). Additionally, users can highlight pieces of information and prompt counterpoints, allowing for a more rich understanding and justification of materials. Other AI tools such as OpenAI ChatGPT can also aid in writing (OpenAI, 2023).

#### Conducting the literature review

A comprehensive literature review is a key component of any systematic review, and must be complete and thorough. It also helps identify gaps that studies can and should address. AI tools streamline this process by allowing authors to rapidly have access to manuscripts that are relevant to their research question. For example, both Consensus.app and Elicit.org allow researchers to ask a research question, and have an AI generated response of papers addressing this question with summaries of information pertinent to the research question (Farid et al., 2022; Ought, 2023). This serves as a helpful starting point of the literature review, and also allows authors to determine if their research question has already been answered in existing literature.

Additionally, understanding background literature can be a burdensome task. AI tools such as Scite.ai finds relevant citations for particular research questions, such as the prevalence of diseases in particular populations (Nicholson et al., 2021). It also scans article references to identify if other papers have supported or refuted any references, which allows rapid understanding of the general consensus on a given topic. Additionally, the Research Rabbit App can suggest related papers based on saved ones in a Zotero library (a bibliographic tool) and LitSuggest offers machine‐learning based recommendations for similar studies based on listed research interests, allowing for more comprehensive understanding of background literature (Allot et al., 2021; Chandra et al., 2023).

When analyzing manuscripts, an AI generated summary may be helpful for screening for information. Indeed, particular AI tools such as PaperDigest, ChatPDF, and OpenAI's ChatGPT can summarize text, and also answer specific questions and extract requested data from text or PDF's (Lichtenberger, 2023; OpenAI, 2023; Wang, 2018).

Referencing relevant articles can be a burdensome task, but can be more efficient with AI tools. For example, the Research Rabbit App allows users to paste a paper title directly into the search bar, which will then suggest a series of related papers ensuring a thorough literature review is conducted based on available evidence (Chandra et al., 2023). It also allows the user to download this and related citations directly to Zotero. This saves needing to download a citation in a particular format, or self‐inputting citations.

### Methods & results

#### Search strategy and eligibility criteria

Formulating a proper search strategy and eligibility criteria is one of the foundational steps in conducting a systematic review. Xtrct provides semantic search capabilities to PubMed searches. In effect, it understands the meaning of a query and finds relevant papers (Maaz, 2023). Users can input the eligibility criteria that they want in natural language, and Xtrct will try to identify the most relevant papers. This allows for a narrower yet more focused search than simple keyword‐based searches as are typical in PubMed. Thalia can be used to search concepts so as to disambiguate words with multiple meanings (e.g., “GAD,” which can refer to generalized anxiety disorder or glutamate decarboxylase based on the context) (Soto et al., 2019).

Wiseone is a browser extension that can help to find other important information sources, by providing cross‐referencing capabilities (Petit et al., 2023). SearchSmart finds the best collection of academic databases, from across 95 databases, for the desired search (Search Smart Research Project, 2023). This helps to maximize the relevance and quality of search results. CitationChaser uses the Lens.org database to automatically construct citation networks for sets of papers. This provides the papers citing or cited by the given paper, which can then be used to identify more articles to include in the review (Haddaway, N R et al., 2021). Consensus.app finds research papers that answer a given question (Farid et al., 2022). PaperDigest provides the most influential research papers for a given topic (Wang, 2018). By clicking on a specific paper, the user can also find related papers, patents, venues, and authors. When collecting literature, these can be used to guide the search.

#### Selection process

The selection process is vital to identifying relevant articles for inclusion. With preset study selection criteria, PRISMA‐compliant systematic reviews typically follow a selection process performed in duplicate (Page et al., 2021). Subsequently, common challenges during this process include high volumes of included studies, and a lack of consistency between reviewers. Both challenges leave screening processes prone to numerous conflicts which can prolong selection and lead to poor quality inclusions. While AI tools cannot entirely replicate a screening process performed in duplicate, they may streamline and assist users.

Covidence and Rayyan.ai provide a platform upon which authors can entirely perform screening and data extraction (Ouzzani et al., 2016; Veritas Health Innovation, 2023). Both tools employ machine learning (a subset of AI) to assist screeners with their selection process. Covidence uses machine learning to incorporate its users behavior and subsequently filter relevant papers. When screeners sort their papers by “most relevant,” papers likely for exclusion are filtered toward the end which significantly streamlines the screening process (Veritas Health Innovation, 2023). Meanwhile, Rayyan parses through imported papers and sorts common terms into four categories based on the PICO (population, intervention, comparison, outcome) model for research questions (Ouzzani et al., 2016). Akin to a search strategy, screeners can use various combinations of these terms to identify relevant papers to optimize their selection process. Notably, both tools' accuracy have yet to be validated in the literature; thus, both require significant user input and cannot entirely replace the screening process. Another tool using AI‐based screening is Distiller SR. Distiller SR, a pay‐per‐use service, offers a validated AI screening that needs to be trained with a small proportion of the hits to be screened (Hamel et al., 2020).

RCT Tagger is a machine‐learning‐powered search engine which identifies human RCTs from PubMed, which is particularly useful in systematic reviews or meta‐analyses that only select RCTs (Cohen et al., 2015). Before studies are imported into a screening platform, users may input their PubMed search strategy to search papers and assign a likelihood of being an RCT as compared to not being an RCT.

#### Data collection process

The data collection process entails reviewing full‐texts and collecting study‐specific information into a standardized form for further synthesis and analysis. While error is mitigated through a process‐en‐duplicate, this may be burdensome as extractors must sift through large amounts of data. Although current AI tools can assist extractors through LLM‐based models, they are not entirely validated and must always be reviewed by extractors to ensure accuracy.

RobotReviewer is an effective tool for initial classification of RCTs. It uses AI to summarize uploaded papers into PICO‐based research questions (Marshall et al., 2016). Users may understand what outcomes are commonly reported among their inclusions which can be used to tailor their extraction forms, or identify high‐yield papers. Furthermore, the PICO‐based summaries can help users group papers with similar populations, outcomes or interventions for meta‐analyses or other collective syntheses.

ChatPDF, HeyGPT, PDF Gear, and Humata AI all work similarly to extract data. Extractors can upload their files in PDF form to their respective tool (Jye, 2023; Khajvandi & Rasmuson, 2023; Lichtenberger, 2023; PDF GEAR TECH PTE. LTD., n.d.). Then, they can chat with the AI program, to ask specific questions about the paper—akin to ChatGPT (OpenAI, 2023). For instance, if reviewers wish to extract the sample size or a specific outcome from a study, they simply need to prompt the program. Should their initial query's accuracy be acceptable, users can ask these programs to extract multiple outcomes into a format that can be copy‐pasted (e.g., CSV format) into a spreadsheet‐based data collection form for further human review and verification.

#### Risk of bias assessment

RobotReviewer also provides a preliminary risk of bias assessment for RCTs, which can streamline users' own assessments (Marshall et al., 2016). As this tools uses Cochrane's risk of bias assessments, reviews using other tools should accordingly correspond RobotReviewer's findings with their tool of choice. Other tools like HeyGPT can be prompted based on parameters set by a risk of bias tool (e.g., NIH, Cochrane) to ask questions about a paper and assess risk of bias (Jye, 2023).

#### Data synthesis

Users can ask ChatGPT to provide guidance with respect to data synthesis methods, specifically on how to analyze specific data types, allowing one to tailor their methods accordingly, and assist data synthesis; however, one must verify with an experienced author since a paucity of data exists with regards to the validity of outputs (OpenAI, 2023). For instance, if a user is interested in performing a stepwise logistic regression in R, ChatGPT will provide the relevant packages, code, and a step‐by‐step tutorial. This is especially useful for users who need extra assistance with programming‐based statistical software or for troubleshooting code facing frequent errors.

### Writing the discussion

The discussion section, as the area of the text where the data and findings are discussed in the context of existing literature, strengths and limitations of the study are described, and clinical relevance of findings are considered, will be considerably less automatable than other parts of the systematic review process.

Scite.ai uses GPT technology to provide users with answers to questions asked in natural language (Nicholson et al., 2021). From there, it provides users with citations to appraise and support their claims. In writing the discussion section, this tool becomes useful as the author tries to appraise the available literature for a specific inquiry. For example, if the author wishes to determine if an association between sex and life expectancy exists, Scite.ai could be used to answer this question while citing the available literature which could be incorporated into the appropriate section of the manuscript (Nicholson et al., 2021).

Jenni.ai similarly provides both writing support in generating sentences relevant to the paragraph being discussed, and also in providing citation suggestions (Park et al., 2019). For the writing of the discussion section, this would increase the speed of writing the text, but also in finding key citations if needed.

ChatGPT, as an LLM with broad use cases, could be used to generate ideas on how to structure the discussion section, to synthesize data points, or to summarize key ideas (OpenAI, 2023). In the discussion section, providing ChatGPT with a summary of the data collected in a meta‐analysis could allow it to write whole paragraphs to interpret and analyze the data as well (OpenAI, 2023).

In the writing of the discussion section, it can often be useful to find the citations used by the included papers to strengthen the arguments discussed. For this purpose, CitationChaser is useful (Haddaway, N R et al., 2021). It finds the lists of references across studies, while also finding lists of articles that cite that study. Once verified, referring to the source citations of a study could then be used to strengthen arguments included in the discussion.

Some tools, such as Consensus.app can be utilized to consider multiple viewpoints and explore the literature (Farid et al., 2022). Consensus.app provides users with findings from multiple articles, with multiple viewpoints. When writing the discussion section, this allows the author to synthesize data, refer to sources that strengthen or weaken the arguments being made, and allows the author to consider multiple perspectives.

Finally, tools like Scholarcy provide the author with a quick summary of research articles selected by the user (Gooch et al., 2023). This can be useful for an author who wants to quickly remind themselves of the highlights or findings of studies included in the systematic review, or to summarize external articles for strengthening the discussion section.

### Risks and limitations of AI tools

Using AI tools, like GPT, in crafting systematic reviews can introduce various risks that can impinge on the accuracy, reliability and credibility of the end product. One such risk is the possibility of AI systems generating information that seems plausible but isn't supported by any real evidence or data. This can be particularly problematic for systematic reviews, which are designed to provide an accurate, objective, and comprehensive synthesis of the existing body of knowledge on a specific topic. If the AI system inserts false information, it can lead to the inclusion of non‐existent studies, false data, or erroneous conclusions in the systematic review, thereby compromising its validity and reliability. This underscores the importance of human oversight and verification.

AI systems can also introduce errors in the process of data summarization. Given that these tools are not capable of understanding context or underlying meanings in the same way humans do, they may inaccurately or inadequately summarize the data they are fed to process. This can result in the misinterpretation of key findings, the omission of crucial details, or the overemphasis of insignificant points. Consequently, these errors can distort the overall message or implications of the systematic review, leading to inappropriate decision making in the application of the review findings.

However, the potential pitfalls of using AI tools in systematic reviews can be mitigated to a significant extent. AI‐based tools that provide citations and sources, such as Elicit, can aid in ensuring the accuracy of the information generated and avoid confabulation (Smith et al., 2023). These tools ensure that the AI output can be traced back to its original source, providing a way to verify the correctness of the synthesized information. This adds a layer of credibility to the information provided by the AI, allowing users to critically appraise the source before accepting the AI‐generated information at face value.

However, users must still apply their expertise to evaluate the information, as even these advanced tools are not immune to errors. For example, we recommend that users are strongly familiar with their statistical analysis program to ensure the feasibility and accuracy of ChatGPT's suggestions (OpenAI, 2023). The combination of AI tools and human expertise can thus harness the power of AI while minimizing its potential risks, resulting in systematic reviews that are both efficient and trustworthy. It is therefore up to the user to be aware of the limitations of the tools they are using, and to critically evaluate the sources provided by the tools to verify their claims. Finally, it is valuable for the user to be aware of which technologies (i.e. GPT) are being used in these AI tools so that they are aware of the limitations underlying the fundamental technology, and to compare tools with similar goals and aims.

As AI tools become increasingly popular, it is essential to improve reporting guidelines regarding AI tools. While AI tools are easy to interpret and use, their coding and methods are extremely heterogeneous, nuanced, and difficult to standardize. Blaizot and colleagues (2021) previously demonstrated in a sample of reviews how AI has extensively been implemented for all stages of a review, especially screening. However, these inferences are made on self‐reporting by the original authors which may be a product of the lack of clear reporting guidelines for AI. Future reporting efforts and guidelines should seek to reconcile complexity with transparency (Blaizot et al., 2022). The development of the Preferred Reporting Items for Systematic Reviews and Meta‐Analyses AI reporting guidelines in healthcare is currently underway, which will serve to support high‐quality, reproducible, and clinically relevant systematic reviews (Cacciamani et al., 2023). Authors should always report all AI tools that have been used at each stage of the evidence synthesis process, the procedures used to ensure quality check, and detail settings and parameters of AI tools to ensure replicability as part of reporting in methods (Bellato, Cristea, et al., 2023). Of note, each tool may have a unique learning curve which may limit efficiency in earlier stages, so benefits may not be seen immediately. Authors must acknowledge the tradeoff between the time to learn each tool and its limitations, and the benefits of efficiency.

Ultimately, are pertinent ethical considerations with using AI tools. Firstly, AI tools that may be accessible are often behind paywalls. Depending on various socioeconomic factors, variable productivity further augmented by such AI models may propagate inequity and fairness in science, as not all researchers may have equal access (Dundar & Lewis, 1998). Furthermore, while balancing transparency with complexity, there is the added prospect of data sharing ethics. Due to the complex and nuanced algorithms, confidentiality of code, and institutional ethics, sharing AI tools may be difficult and reproducibility could be impacted (Gallifant et al., 2022). Transparency is necessary as it allows for improvement of models and evolution of methods; however, depending on how patient information is used, AI models may compromise confidentiality (Gallifant et al., 2022). Nevertheless, the secondary nature of systematic reviews and integrative research mitigates that aspect of confidentiality. Finally, depending on how LLMs are used, there is a question of attributing authorship with respect to accountability and responsibility for the information produced. Due to their lack of self‐determination, AI tools fulfill neither category with respect to authorship. Therefore, if AI tools are to produce erroneous information, as aforementioned they often do, blame cannot be deferred and ultimately, the burden of correcting such errors comes upon the authors themselves. However, as AI rapidly evolves, these contentions may have to evolve accordingly as well (Hosseini et al., 2023). There are also concerns with regards to scientific integrity if researchers are using AI tools without acknowledging and reporting this. These ethical considerations are not exhaustive in nature and future studies should continue to investigate and improve AI use guidelines in systematic reviews to ensure research is ethically performed at the highest quality.

## CONCLUSION

In conclusion, the integration of AI tools into the systematic review process has considerable potential to improve efficiency and streamline the research workflow. Despite their potential benefits to efficiency and comprehensiveness, it is important to remember that these tools should only ever be guided from and used as a supplement to human content and clinical insight, quality check, manual review, writing, and evaluation. At each stage of the systematic review process, authors are obligated to cross‐verify the information provided by these tools, ensure the relevance and quality of the included papers, and apply their expert judgment to interpret and synthesize these findings. Caution must also be exercised to ensure tools aren't used in a manner that reinforce authors pre‐conceptions through preferential bias in acquiring information based on users command. AI tools, when combined with human expertise, have the potential to contribute to an efficient, comprehensive, and impactful systematic review. Further research is needed to evaluate the effectiveness, accuracy, validity and limitations of these AI tools, and to assess their impact on the quality and reliability of research synthesis. It is also of utmost importance that these tools be appropriately cited in the methods section of the paper for full transparency.

## AUTHOR CONTRIBUTIONS

**Nicholas Fabiano**: Conceptualization; data curation; investigation; methodology; project administration; resources; supervision; validation; visualization; writing – original draft; writing – review & editing. **Arnav Gupta**: Conceptualization; data curation; methodology; project administration; resources; supervision; validation; writing – original draft; writing – review & editing. **Nishaant Bhambra**: Conceptualization; investigation; methodology; project administration; resources; writing – original draft; writing – review & editing. **Brandon Luu**: Conceptualization; methodology; writing – original draft; writing – review & editing. Stanley Wong: Conceptualization; data curation; methodology; resources; writing – original draft; writing – review & editing. **Muhammad Maaz**: Conceptualization; methodology; writing – original draft; writing – review & editing. **Jess G. Fiedorowicz**: Conceptualization; methodology; project administration; resources; supervision; writing – original draft; writing – review & editing. **Andrew L. Smith**: Methodology; supervision; writing – original draft; writing – review & editing. **Marco Solmi**: Conceptualization; methodology; project administration; resources; supervision; validation; writing – original draft; writing – review & editing.

## CONFLICT OF INTEREST STATEMENT

MM developed xtrct.app which was cited in this paper. MS received honoraria/has been a consultant for Abbvie, Angelini, Lundbeck, Otsuka, unrelated to this work.

## ETHICS STATEMENT

Research ethics board approval for this type of research is waived at the University of Ottawa.

## Data Availability

Data sharing is not applicable to this article as no new data were created or analyzed in this study.
